# Osseous Union after Jaw Reconstruction with Fibula-Free Flap: Conventional vs. CAD/CAM Patient-Specific Implants

**DOI:** 10.3390/cancers14235774

**Published:** 2022-11-24

**Authors:** Michael Knitschke, Magdalena Yonan, Fritz Christian Roller, Jörn Pons-Kühnemann, Sameh Attia, Hans-Peter Howaldt, Philipp Streckbein, Sebastian Böttger

**Affiliations:** 1 Department of Oral and Maxillofacial Surgery, Justus-Liebig-University, Klinikstrasse 33, 35392 Giessen, Germany; 2 Department of Diagnostic and Interventional Radiology and Pediatric Radiology, Justus-Liebig-University, Klinikstrasse 33, 35392 Giessen, Germany; 3 Medical Statistics, Institute of Medical Informatics, Justus-Liebig-University Giessen, 35392 Giessen, Germany

**Keywords:** virtual surgical planning, 3D printing, 3D technology, three-dimensional, osteotomies, non-union, malunion, patient-specific implant, CAD/CAM

## Abstract

**Simple Summary:**

After jaw reconstruction using a fibula-free flap (FFF), an incomplete osseous union is a complication that significantly lengthens the period until the dental rehabilitation and affects the patients’ morbidity and quality of life. Patients-specific implants (PSI) are now widely used in reconstructive jaw surgery to optimize surgical procedures and lower the rate of complications. This study aims to determine the probability of osseous union following FFF jaw reconstruction with respect to the use of PSI or conventional osteosynthesis (non-PSI). Additionally, risk factors for an incomplete osseous union were determined.

**Abstract:**

This is a monocentric, retrospective study of patients who underwent successful immediate or delayed maxilla or mandible reconstructions with FFF from January 2005 to December 2021. Panoramic radiograph, computed tomography scans, and cone-beam CTs were analyzed concerning the osseous union of the intersegmental junctions between maxillary or mandibular native jaw and fibular bone. The primary parameter was to estimate the status of osseous union according to osteosynthesis type. A total number of 133 patients (PSI: *n =* 64, non-PSI: *n* = 69) were included in the present study. The mean age was 56.7 ± 14.0 (Range: 14.7–82.7); the primary diagnosis was in 105 patients a malignant (78.9%) and in 20 patients a benign (15.0%) tumor. Mandible reconstruction was performed on 103 patients (77.4%), and on 30 patients (22.6%), maxilla reconstruction was performed. The radiographic images provided a rate of incomplete osseous union (IOU) of about 90% in both groups in the first 6 months. Imaging between 6 and 12 months reveals an IOU rate in the non-PSI group of 46.3% vs. 52.5% in the PSI group, between 12 and 24 months, an IOU rate of 19.6% vs. 26.1%, between 24 and 36 months 8.9% vs. 21.7%, and after 36 months the IOU rate decreases to 4.2% vs. 18.2%. Multivariate logistic regression shows that only osteosynthesis type (OR = 3.518 [95%-CI = 1.223–10.124], *p* = 0.02) and adjuvant radiotherapy (OR = 4.804 [95%-CI = 1.602–14.409], *p* = 0.005) are independent risk factors for incomplete osseous union. Cox regression revealed that the variables plate-system (Hazard ratio, HR = 5.014; 95 %-CI: 1.826–3.769; *p* = 0.002) and adjuvant radiotherapy (HR = 5.710; 95 %-CI: 2.066–15.787; *p* < 0.001) are predictors for incomplete osseous union. In our study, the rate of incomplete bony fusion was significantly higher in the PSI group. Jaw-to-fibula apposition zones were significantly more affected than intersegmental zones. In multivariate analysis, a combination of osteosynthesis with PSI and adjuvant radiotherapy could be identified as a risk constellation for incomplete ossification.

## 1. Introduction

The free fibula flap (FFF) is the preferred osteo-cutaneous flap to reconstruct jaw defects [1]. Its length and height allow prosthetic rehabilitation with dental implants in a one- or two-staged procedure and a considerable functional and aesthetic treatment outcome [2,3]. Alternatively, microvascular grafts of the iliac crest or the scapula were often used as flaps for microvascular reconstruction of the osseous continuity of the jaws [4,5]. Disadvantages are the short vascular pedicle and the limitations in microvascular anastomosis [6]. The best reconstruction restores the patient’s appearance and ability to talk, swallow, masticate, and maintain a patent airway without needing a tracheostomy [7]. It also results in a normal facial profile and occlusion [8]. Modern microvascular reconstructive treatments enable the patient to finally return to an oral diet, to speak clearly, and to integrate back into society with the help of adequate dental and functional rehabilitation [2,3,9]. 

However, nowadays, virtual surgical planning and the application of patient-specific implants (PSI) present a popular method for jaw reconstruction with microvascular FFF [10,11]. The described advantages include a shortened surgical time, achievable precision, and predictable accuracy of a bony bearing in the context of implantology, especially in three-dimensional alignment for maxillary reconstruction [12,13]. While complication rates for virtually planned jaw reconstructions with PSI were stable in a systematic review [12], individual cohort studies show decreased bony non-union rates [14]. 

In contrast, other studies revealed significant differences in the osseous union of mandible-to-fibula and intersegmental junction when PSI was used to stabilize mandible reconstruction. They reported an increased rate of incomplete osseous union after around 12 months in the CAD/CAM-PSI group between 35.6 and 45.9% in comparison to a conventional osteosynthesis group with 13.6–33.0% [15,16]. In literature, the rate for incomplete bony union for different bone flap types ranges from 5 to 26.3% [14,17,18,19,20]. Influencing factors are the number of segments, type of osteosynthesis and reconstruction procedures, and pre- and/or post-reconstructive radiotherapy [21,22,23]. The previous investigations have a common weakness: basing their observation on a single radiological scan 12 months after reconstruction to assess bony fusion [15,16]. This time seems appropriate when complete ossification has occurred, however, it is unclear how incomplete ossification progresses over time. For the period when the complete bony fusion of free bone flap segments for jaw restoration can be anticipated, available data is insufficient in current literature. But this is of major interest if rehabilitation with dental implants is contemplated. There are some findings which indicate that when PSI is utilized instead of conventional osteosynthesis, the rate of osseous union is decreased. This retrospective study investigates this issue by evaluating all available radiographic data to determine the time to ossification with conventional (non-PSI) osteosynthesis procedures compared to CAD/CAM-PSI. The primary parameter was to estimate the status of osseous union according to osteosynthesis type. Furthermore, it will analyze which factors this ossification depends on and which clinical relevance arises for the patients. 

## 2. Materials and Methods

The monocentric, retrospective study enrolled patients who underwent immediate and delayed maxilla and mandible reconstructions with FFF from January 2005 to December 2021. Panoramic radiograph (OPT), computed tomography (CT) scans, and cone-beam CTs (CBCT) were analyzed concerning the osseous union of the intersegmental junctions between the maxillary or mandibular bone and fibula graft, and between bi- or tri-segmental fibula reconstructions. Osteosynthesis of jaw reconstruction was performed either with conventional plates (non-PSI) or patient-specific implants (PSI) after virtual surgical planning (VSP). Laser-melted CAD/CAM-PSI titanium plates with a thickness of 2.0 to 2.5 mm (KLS Martin, Tuttlingen, Germany) were compared with conventional osteosynthesis in terms of osseous union. PSI were used as continuous plates, whereas conventional plates were positioned segmentally. Cutting guides were employed only in the PSI group for flap harvesting at the donor site and for marking the resection planes and drill holes at the recipient site. The conventional group harvested fibula segments and shaped them using freehand osteotomies. Both conventional Unilock 2.0 and PSI were anchored in the mandibula using bi-cortical locking screws. The surgeon chose whether to use locking or non-locking screws for plate fixation to the FFF intraoperatively. Since 2015, all jaw reconstructions had been planned virtually and stabilized with PSI.

### 2.1. Inclusion and Exclusion Criteria for Study Subjects 

The inclusion criteria were defined as: (a) successful reconstruction of the mandibula or maxilla, (b) at least two available OPT-, CBCT-, or CT-scans of the jaw over the entire follow-up time, (c) medical records and operative reports. Patients were excluded from the study if X-ray or medical records were not accessible. 

### 2.2. Study Parameters and Evaluator Calibration

**Figure 1:**
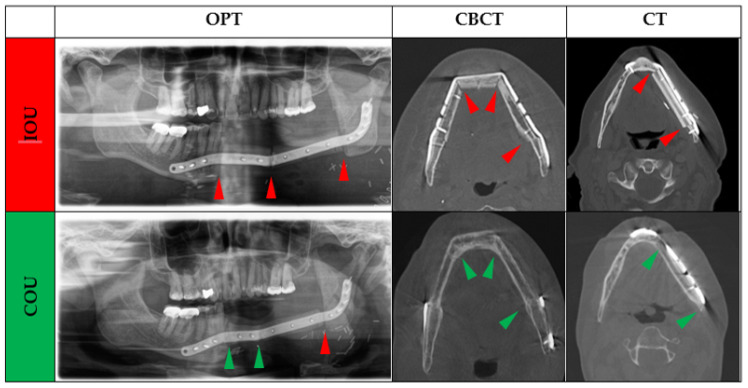
All available radiographs (OPT, CT, and CBCT-scans) were analyzed independently for ossification of each gap by the two authors. In OPT, ossification was defined as incomplete (IOU) if the interosseous gap appeared less than 50% ossified or as complete (COU) if it appeared more than 50% ossified by visual assessment. In axial CBCT or CT imaging, IOU was assigned if the two segments didn’t show fusion of the callus, marrow, or cortices, if there was a persistent gap between segments, or partial bridging between adjacent bone cortices, or marrow was noted. If the matching cortices were linked without major gaps, COU was given. Note: Green arrow: complete osseous union (COU); Red arrow: incomplete osseous union (IOU).

The patients’ medical records were evaluated according to the used plate system and assigned to the non-PSI or patient-specific (PSI) osteosynthesis group for fixation of jaw reconstruction. The dentition status of the diseased jaw and the healthy opposing jaw were recorded separately and classified at the time of reconstruction: (i) Complete (ii) Partially edentulous, or (iii) Edentulous. The following parameters were collected: age at flap transfer, sex, primary disease, number of used fibula segments, and ossification status of each junction zone native bone-to-fibula (Jaw-to-fibula, J-F) or between fibula segments (Fibula-to-fibula, F-F). The grade of osseous union was classified as complete (COU) or incomplete osseous union (IOU). In OPT, ossification was defined as incomplete (IOU) if the interosseous transition zone was less than 50% ossified, or complete (COU) if it was more than 50% ossified by visual assessment. The observer was trained on representative OPTs, CBCT, and CT-scans for an accurate evaluation of bony fusion’s status (Figure 1). Axial imaging was used for evaluation in CBCT and CT. If early callus development, persistent gap between segments, or subtotal bony bridging between adjacent bone cortices or marrow was noted, IOU was assigned. If the matching cortices were linked without major gaps, COU was given. Two investigators independently analyzed each gap (MY and MK). Any disagreements between the two authors were discussed and judged by a third author (FR), who is a radiologist. 

### 2.3. Statistical Analyses

Continuous variables were reported using mean, standard deviation, median, and interquartile interval (Q1, Q3). Categorical data were recorded as frequencies and percentages. The bivariate analysis included Student’s t-test to compare continuous quantitative variables between both groups (Non-PSI vs. PSI). Chi-square and Fisher’s tests were performed for categorical variables. Binary logistics was conducted to identify risk factors for incomplete bony fusion. Only statistically significant independent risk factors for incomplete osseous union were used for multivariate analysis. Cox regression statistics were performed to calculate the probability of osseous union for both osteosynthesis types regarding time after jaw reconstruction. Cohen’s Kappa (κ) statistics were calculated to assess the interobserver reliability between MY and MK. A value of *p* ≤ 0.05 was defined as statistically significant. The statistical analysis was performed in collaboration with the Institute of Medical Informatics of Justus Liebig University Giessen using SPSS software version 28 (SPSS Inc., Chicago, IL, USA). 

### 2.4. Ethics Statement/Confirmation of Patients’ Permission

The Ethics Committee of Justus-Liebig University Giessen, Faculty of Medicine, approved the study (AZ103/22 on 11.07.2022). Patients’ permission/consent was not necessary for this retrospective study. The patients agreed that their X-ray images could be used anonymously in the publication. 

## 3. Results

A total of 169 patients underwent successful FFF reconstruction of the jaw, and 133 fulfilled the chosen inclusion criteria and were included in the present study. A number of cases (*n* = 36) had to be excluded due to failed inclusion criteria.

The entire sample characteristics and those categorized concerning the used kind of osteosynthesis are summarized in Table 1: mean age was 56.7 ± 14.0 (Range: 14.7–82.7); 83 patients were male (62.4%) while 50 (37.6%) were female. Primary diagnosis was in 105 patients a malignant (78.9%) and in 20 patients a benign (15.0%) tumor. The reconstruction of the maxilla was performed in 30 patients (22.6%) and the reconstruction of the mandible in 103 patients (77.4%). Surgery only was the treatment of choice in 72 patients (54.1%), and adjuvant radio(chemo)therapy was performed in 61 patients (45.9%). 

**Figure 2:**
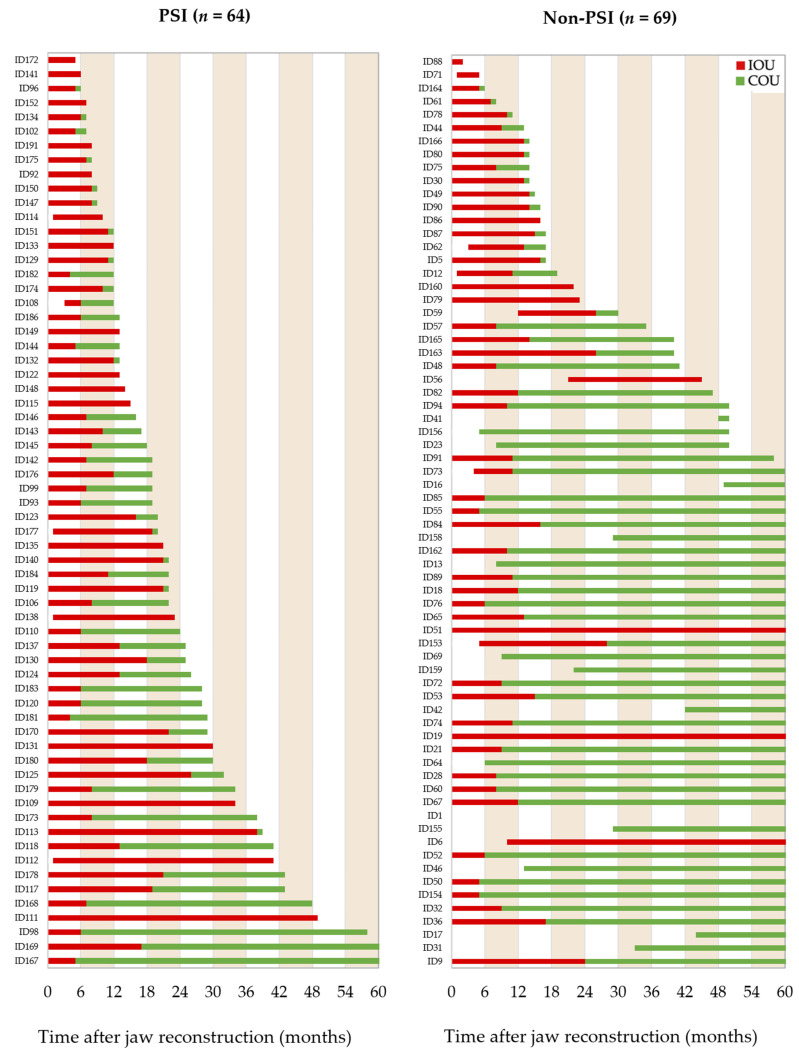
The bar charts show the ossification of junction zone between free flap segments or flap segments and native bone on patient level (ID) based on reviewable X-rays (OPT, CT, CB-CT) categorized using osteosynthesis type. In OPT, ossification was defined as incomplete (IOU) if the interosseous transition zone was less than 50% ossified, or complete (COU) if it was more than 50% ossified. In axial CBCT or CT imaging, IOU was assigned if early callus development, persistent gap between segments, or subtotal bony bridging between adjacent bone cortices or marrow was noted. If the matching cortices were linked without major gaps, COU was given. For a status change from “incomplete” (IOU) to “complete osseous union” (COU) all transition zones had to be judged as complete ossified. On the X-axis, the follow-up is given in months.

All available radiographic images were reviewed for the status of osseous union of the junction zones and categorized concerning the used osteosynthesis type. The status of incomplete (red) and complete osseous union (green) were plotted in bar charts related to the follow-up interval (Figure 2). The drawn follow-up interval was scaled to 60 months for better comparability of the groups in the figure. 

**Figure 3:**
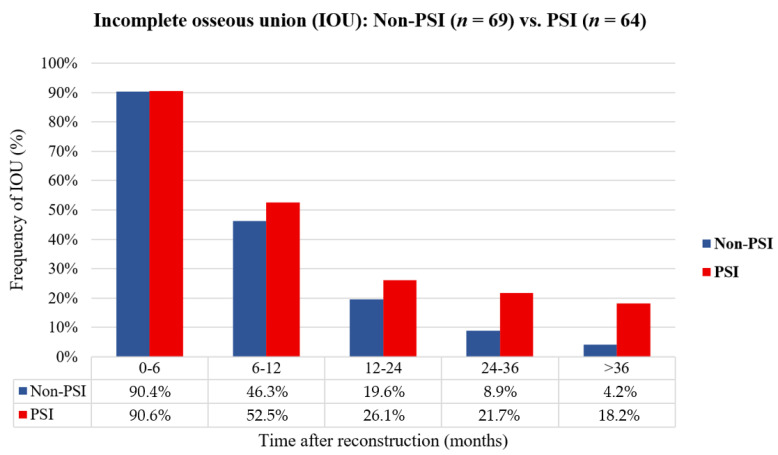
The bar chart shows the comparison of osteosynthesis methods non-PSI vs. PSI regarding the relative proportions of at least one incompletely ossified (IOU) junction per patient clustered by time interval post jaw reconstruction with a fibula flap (months).

Interobserver reliability was obtained for the graduation of osseous union. Cohen’s Kappa value of 0.981 indicated a good match between the observers. For further evaluation, time intervals after reconstruction were defined and the rate of incomplete ossified (IOU) junctions per patient were determined. Ossification was considered incomplete if at least one connection between the flap segments or a segment and the native bone was incomplete (Figure 3). Our results show that the rate of IOU was higher in the CAD/CAM-PSI group than in the conventional group. The radiographic images provided a rate of incomplete osseous union of about 90% in both groups in the first six months. Imaging between 6 and 12 months reveals an IOU rate in the non-PSI group of 46.3% vs. 52.5% in the PSI group, between 12 and 24 months, an IOU rate of 19.6% vs. 26.1%, between 24 and 36 months 8.9% vs. 21.7%, and after 36 months the IOU rate decreases to 4.2% vs. 18.2%.

**Figure 4:**
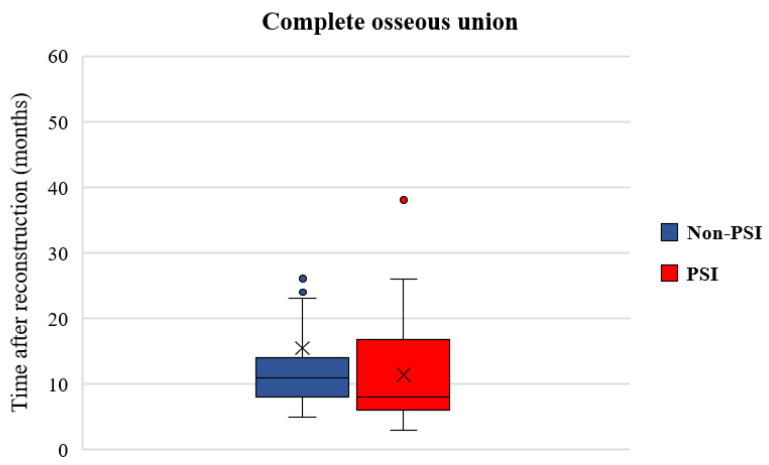
Boxplot shows the time of recorded radiologically switch from incomplete to complete osseous union on patient level (non-PSI: mean ± SD = 15.4 ± 20.9 months, median = 11.0 months; PSI: mean ± SD = 11.4 ± 7.0 months, median = 8.0 months; *p* = 0.210). Note: ×: mean.

In total, 105 junctions between free flap segments (F-F) (Non-PSI: *n* = 45 vs. PSI: *n* = 60) and 227 junction zones between flap segments and native bone (J-F) (Non-PSI: *n* = 126 vs. PSI: *n* = 101) in 133 patients were evaluated. On radiographic images (OPT, CT, CB-CT), 292 junctions were evaluated as complete ossified (COU) (COU rate patient level: 82.7%, COU rate junction level: 88.0%), whereas 40 had an incomplete osseous union (IOU rate patient level: 17.3%, IOU rate junction level: 12.0%). The median period from surgery to the first scan was 4 months (range 4–120 months). The status change from incomplete to complete osseous union on radiographic imaging took a mean time of 16 months (range 4–143 months). In comparison to the used osteosyntheses systems (Figure 4) data reveals that osseous union becomes earlier visible in the CAD/CAM-PSI than in the non-PSI group (non-PSI: median: 11.0, mean 15.4 ± 20.9, range 5–143 months vs. PSI: median: 8.0, mean 11.4 ± 7.0, range 4–38 months; *p* = 0.210). The Cox model also confirmed this finding, but the difference was without statistical significance.

**Figure 5:**
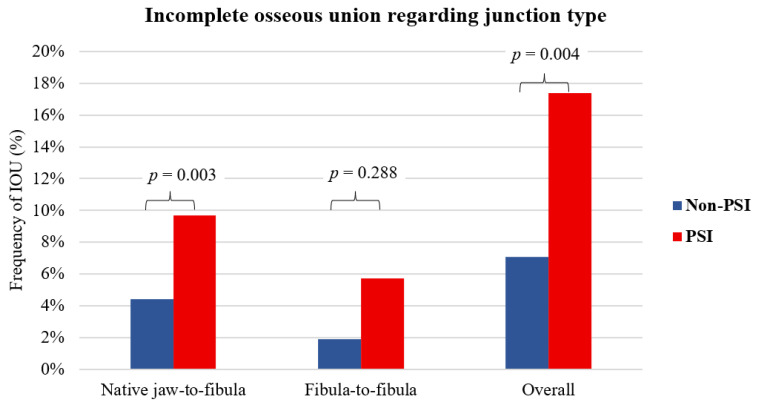
The bar chart shows the relative frequency of incomplete osseous union (IOU) on junction level grouped by osteosynthesis type non-PSI vs. PSI on the last radiographic imaging. There were 32 IOU for 227 appositions (non-PSI: *n* = 126 vs. PSI: *n* = 101) between native jaw-to-fibula, yielding a 14.1% total incomplete union rate (non-PSI: *n* = 10, 4.4% vs. PSI: *n* = 22, 9.7%; χ^2^ (1, *n* = 227) = 8.8749, *p* = 0.003). There were 8 IOU for 105 appositions (non-PSI: *n* = 45 vs. PSI: *n* = 60) between osteotomized neighboring free flap segments, yielding a 7.6% total incomplete-union rate: (non-PSI: *n* = 2, 1.9% vs. PSI: *n* = 6, 5.7%; χ^2^ (1, *n* = 105) = 1.1276, *p* = 0.288). This corresponds to an IOU rate in the non-PSI of 7.1% (12 out of 171) and PSI 17.4% (28 out of 161); χ^2^ (1, *n* = 332) = 8.4215, *p* = 0.004 for all junctions.

The final scan revealed 292 (90.4%) junctions with complete osseous union and 40 (9.6%) with incomplete union. The average time until the final scan was 54 months (range 2–216 months). In 227 appositions between native bone and free fibula flap segments, 32 incomplete bony connections were observed, giving an overall rate of 14.1% for incomplete ossified connections (non-PSI: *n* = 10, 4.4% vs. PSI: *n* = 22, 9.7%; *p* = 0.003). In 105 appositions between adjacent free flap segments, 8 incomplete bony connections were observed, resulting in an overall rate of 7.6% incomplete ossified connections (7.6% incomplete-union rate; non-PSI: *n* = 2, 1.9% vs. PSI: *n* = 6, 5.7%; *p* = 0.288). This corresponds to an IOU rate in the non-PSI of 7.1% (12 out of 171) and PSI of 17.4% (28 out of 161); χ^2^ (1, *n* = 332) = 8.4215, *p* = 0.004 for all junctions (Figure 5).

**Table 2:** Univariate analysis of assessed factors on complete (COU) vs. incomplete osseous union (IOU) on last X-ray. DS, dental status; SD, standard deviation; OR, Odds-Ratio; CI, confidence interval.

Parameter		COU, *n* (%)	IOU, *n* (%)	*p*-Value	OR [95%-CI]
Age, years (Mean ± SD)		55.9 ± 14.6	60.6 ± 9.5	0.143	1.028 [0.991; 1.067]
Gender	Male	67 (60.9)	16 (69.6)	0.430	1.467 [0.558; 3.859]
	Female	43 (39.1)	7 (30.4)		
Reconstruction site	Maxilla	26 (23.6)	4 (17.4)	0.517	1.470 [0.459; 4.711]
	Mandibula	84 (76.4)	19 (82.6)		
Osteosynthesis type	Non-PSI	63 (57.3)	6 (26.1)	0.006	3.798 [1.391; 10.370]
	CAD/CAM-PSI	47 (42.7)	17 (73.9)		
ASA ≥ 3	Yes	44 (40.0)	11 (47.8)	0.490	1.375 [0.558; 3.391]
	No	66 (60.0)	12 (52.2)		
Tobacco	Yes	60 (54.5)	17 (73.9)	0.093	2.361 [0.866; 6.441]
	No	50 (45.5)	6 (26.1)		
Alcohol	Yes	33 (30.0)	13 (56.5)	0.018	3.033 [1.209; 7.610]
	No	77 (70.0)	10 (43.5)		
Fibular segments	1	42 (38.2)	7 (30.4)		
	2	44 (40.0)	13 (56.5)	0.267	1.773 [0.645; 4.874]
	3	24 (21.8)	3 (13.0)	0.696	0.750 [0.177; 3.173]
Adjuvant radiotherapy	Yes	43 (39.1)	18 (78.3)	0.001	5.609 [1.939; 16.227]
	No	67 (60.9)	5 (21.7)		
Composite flap	Yes	87 (79.1)	20 (87.0)	0.391	1.762 [0.481; 6.451]
	No	23 (20.9)	3 (13.0)		
DS reconstruction site	Complete	19 (17.3)	4 (17.4)		
	Partially	75 (68.2)	11 (47.8)	0.571	0.697 [0.200; 2.432]
	Edentulous	16 (14.5)	8 (34.8)	0.217	2.375 [0.602; 9.367]
DS non-reconstruction	Complete	21 (19.1)	2 (8.7)		
site	Partially	66 (60.0)	12 (52.2)	0.452	1.909 [0.395; 9.226]
	Edentulous	23 (20.9)	9 (39.1)	0.092	4.109 [0.795; 21.232]
Plate exposure	Yes	23 (20.9)	8 (34.8)	0.158	2.017 [0.762; 5.340]
	No	87 (79.1)	15 (65.2)		
Screw loosening	Yes	13 (11.8)	6 (26.1)	0.083	2.633 [0.880; 7.880]
	No	97 (88.2)	17 (73.9)		

**Figure 6:**
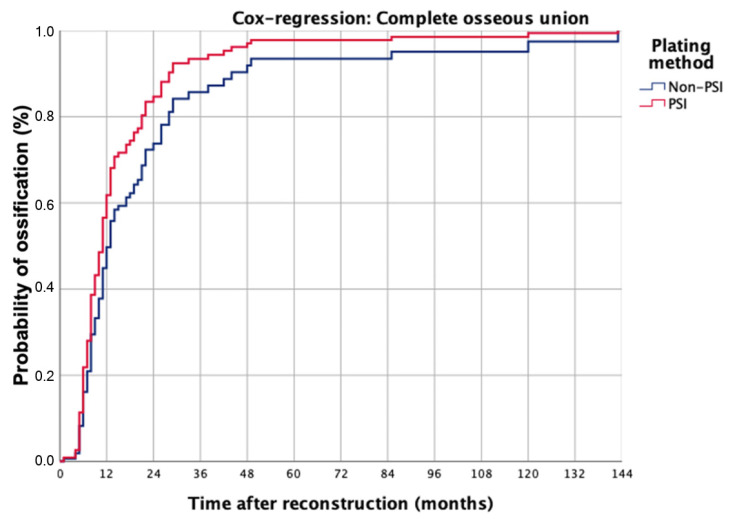
The Cox regression model was calculated for osseous union after jaw reconstruction, separated regarding used osteosynthesis systems, and included categorical covariates of osteosynthesis type and chosen therapy.

Univariate analysis revealed that the parameters of osteosynthesis type (OR = 3.798 [95%-CI = 1.391–10.370], *p* = 0.006), alcohol abuse (OR = 3.033 [95%-CI = 1.209–7.610], *p* = 0.018) and adjuvant radiotherapy (RCT) (OR = 5.609 [95%-CI = 1.939–16.227], *p* = 0.001) are independent risk factors for incomplete osseous union. Multivariate logistic regression shows that only osteosynthesis type (OR = 3.518 [95%-CI = 1.223–10.124], *p* = 0.02) and adjuvant radiotherapy (OR = 4.804 [95%-CI = 1.602–14.409], *p* = 0.005) are independent risk factors for incomplete osseous union (Table 3). The model was significant in the Omnibus-Test (*p* < 0.001; Nagelkerkes R^2^ = 0.248). For further analysis, the parameters of osteosynthesis type and therapy were used as categorial covariates and categorized as complete or incomplete bony fusion in the Cox-regression model (Figure 6). 

No significant difference for both plating methods was recorded when complete bony fusion (COU) occurred (*p* = 0.262). Differences for plating type PSI vs. Non-PSI were significant when incomplete osseous union (IOU) was found (*p* < 0.001).

The Cox regression model for complete osseous union (COU) shows that this occurred in both groups in 50% of the cases at 12 months. 75% occurred after 13 months in the PSI group and 24 months in the non-PSI group. This difference was not statistically significant (*p* = 0.262). For incomplete osseous union the model describes that 20% of incomplete bony fusions occurred at 12 months in the PSI group and 50% at about 24 months, while 4% occurred at 12 months and 15% at about 24 months in the non-PSI group (*p* < 0.001). Cox regression analysis revealed that the variables plate system (Hazard ratio, HR = 5.014; 95%-CI: 1.826–3.769; *p* = 0.002) and adjuvant radiotherapy (HR = 5.710; 95%-CI: 2.066–15.787; *p* < 0.001) are predictors for incomplete osseous union. 

Complications after jaw reconstruction are summarized in Table 4. Complications associated with incomplete bony fusion (IOU) were observed in Non-PSI and PSI groups. The PSI group found significant differences between osteonecrosis (*p* = 0.016) and screw loosening (*p* = 0.048). Plate exposure was recorded in more than 20% of cases in both groups, yet this did not affect complete bony fusion (COU). Screw loosening was associated with IOU in 29.4% of cases in the PSI group (*p* = 0.048).

## 4. Discussion

Following fibula-free flap reconstruction of the jaws, ideal healing is represented by osseous union at the neo-jaw site. Bone union offers stability and strength for dental rehabilitation or to prevent pathologic fractures from occlusive loads. This study aimed to identify patient and surgery-associated parameters that can affect the bony fusion after jaw reconstruction with a vascularized fibula-free flap. Furthermore, the time required for a complete ossification should be determined. A slightly earlier radiologically visible ossification in PSI than in the non-PSI group was found (non-PSI: median: 11.0, mean 15.4 ± 20.9, range 5–143 months vs. PSI: median: 8.0, mean 11.4 ± 7.0, range 4–38 months; *p* = 0.210). This finding must be interpreted carefully due to the limitations of the retrospective study and the non-standardized time of imaging and different types of images for assessment. 

In this retrospective study, OPT images were obtained more frequently than CT and CBCT images during follow-up. The OPT image is suitable for basic, qualitative assessment of ossification in the context of mandibular reconstructions [24]. A quantitative assessment is not possible with OPT. For this purpose, high-resolution CBCT can be used since it allows complete visualization of the osteotomy gaps. Artifacts due to the osteosynthesis plates can have a quality-reducing effect [25]. CBCT has been used especially for planning dental rehabilitation in our patient pool. CT scans were acquired for oncological follow-up and in the past with a slice thickness of up to 3 mm. In the meantime, the slice thickness has decreased significantly, which allows a better assessment of the segmental gaps. For reasons of radiation protection, the indication for CT examinations after reconstruction in benign diseases is very strict, so it was not run routinely.

A significantly higher rate of incomplete osseous union occurred in the PSI group (IOU rate at patient level: PSI: 26.6% vs. non-PSI: 8.7%; *p* = 0.006; IOU rate at junction level: PSI: 17.4% vs. non-PSI: 7.1%; *p* = 0.004). With limitations of the retrospective study design, our data demonstrate that if CAD/CAM-PSI was used, a higher rate of incomplete bony fusion between native bone and fibula flap bone (*p* = 0.003) was found than between the flap bone segments in polysegmental reconstructions (*p* = 0.288). Hashemi et al. [26] analyzed 38 osteotomy sites in *n* = 13 patients after jaw reconstruction with FFF and found similar results. They discovered that the internal and middle gap locations did not independently approach statistical significance. With limitations to the small sample size, their findings suggest that the jaw-to-fibula junction is more susceptible to variations in gap widths due to their closeness to the reconstruction bar, whereas the interior and middle gap sites may have a greater threshold for variability [27]. Mechanical properties of PSI are assumed to be causative for lower physiological stimulation of bone remodeling [15,16,28]. In our sample, the dentition status of the reconstructed “neo-jaw” and the healthy (non-affected) jaw had no impact on the presence of incomplete ossification (Table 2). However, the influence of functional factors on physiological bone remodeling is repeatedly emphasized in studies [15,29]. It is striking that biomechanical influences from the side of the reconstruction as well as gender also had no significant effect on the ossification status in the present study. 

Previous analysis found that patient-specific implants (PSI) for osteosynthesis of the mandible were associated with an increased rate of incomplete osseous union after approximately 12 months [15,16]. An influence of used osteosynthesis type on the time of radiologically observed ossification of the junctions between jaw-to-fibula and intersegmental (fibula-to-fibula) was also found and confirmed by the present study.

On the one hand, ossification was detected slightly earlier in the CAD/CAM-PSI group compared to the non-PSI group. The high accuracy of fit and small gap width contributes to this. Some authors report that gap widths broader than 1 mm are unlikely to close completely [30], while others suggest that gap widths larger than 2.55 mm are related to a higher risk of non-union [26]. Optimal gap width smaller than 1 mm has been reported as an optimal environment for bony fusion [30,31]. But keep in mind, that primary bone healing is not possible after microvascular jaw reconstruction, because neither the necessary gap width <0.01 mm for ideal contact healing [32] nor <0.8–1 mm for primary gap healing can be achieved [33]. Secondary bone healing can occur under good conditions in broader gaps [34]. Bony fusion of the fibula segments to the jaw bone is similar to fracture healing and undergoes a gradual remodeling into new bone, which transforms the callus into a mature bony union [35]. The development of a callus formation is a crucial indicator and predictor of fracture union [36]. While primary bone healing takes about 6 months, secondary bone healing can take more than a year [37]. Highly precise anatomical reduction or rigid stability is not crucial for secondary bone repair [31]. Bone healing is enhanced by weight-bearing (e.g., occlusive forces or for dental rehabilitation) and micromotion. However, a non-union or even a delay in healing has been connected to excessive motion and/or load [38]. Secondary bone healing frequently occurs when comminuted fractures are treated non-operatively or with some surgical techniques that allow some movement at the fracture site, like external fixation or internal fixation devices [39,40]. Especially if microvascular free flap reconstruction in patients becomes necessary, predominantly patients suffer from a malign tumor or the consequences of adjuvant oncological therapy (osteoradionecrosis, medication-related osteonecrosis of jaw). Patients are typically older [41,42,43], and often have cardio-vascular comorbidities, in addition to the risk factors of tobacco and alcohol consumption [22,44,45]. Swendseid et al. [30] state, that the appearance of partial union or non-union on a routine scan may be a good indicator of future wound problems and that the best segment apposition should be performed [30]. In a retrospective study including 102 patients, West et al. [46] showed that smoking history, the number of osteotomies, and flap nonviability are all related to plate exposure following mandibular reconstruction. They claimed that long-term results following mandibular reconstruction could be enhanced by reducing surgical problems and performing fewer osteotomies to prevent overly small flap bone segments. Therefore, the number of osteotomies was a risk factor for complications after a fibula-free flap [26,46], which may contribute to partial flap loss [47].

Plate exposure was found with 23.3% in the present investigation (PSI: *n* = 15, 23.4% vs. non-PSI: *n* = 16, 23.2%; χ^2^ (1, *n* = 133) = 0.0012, *p* = 0.973). It was identified as a statistically significant risk factor for incomplete osseous union in a previous study [16]. Its incidence is reported between 3 and 46% [48,49], and on average with 20% [50,51,52] for conventional plates and 29.7% for CAD/CAM-PSI [15]. When this complication arises, it is associated with high morbidity and expense, necessitates more hospital stays, increases antibiotic use to avoid infection, and may necessitate further surgical intervention [53]. Sobti et al. [54] reviewed the current literature complications regarding the use of mini-plate versus reconstruction bars for fixation after jaw reconstruction with fibula flap. They found higher rates of plate-related complications (32.5% vs. 18.8%, *p* < 0.01, respectively), fistula formation (15.8% vs. 4.7%, *p* = 0.04), total flap loss (9.4% vs. 4.7%, *p* = 0.02), partial flap loss (20.6% vs. 6.1%, *p* < 0.01) in the mini-plate group. No differences were assessed according to wound infection, and mal-union/non-union between the osteosynthesis groups [54]. The authors concluded that using mini-plates may increase the risk of difficulties compared to reconstruction bars [54]. Contrary to our own preliminary investigations on parts of the study population [16] this evaluation showed that exposed osteosynthesis material did not reach statistical significance as an independent risk factor for IOU in univariate analysis (OR = 2.017, 95%-CI = 0.762–5.340; *p* = 0.158). 

**Figure 7:**
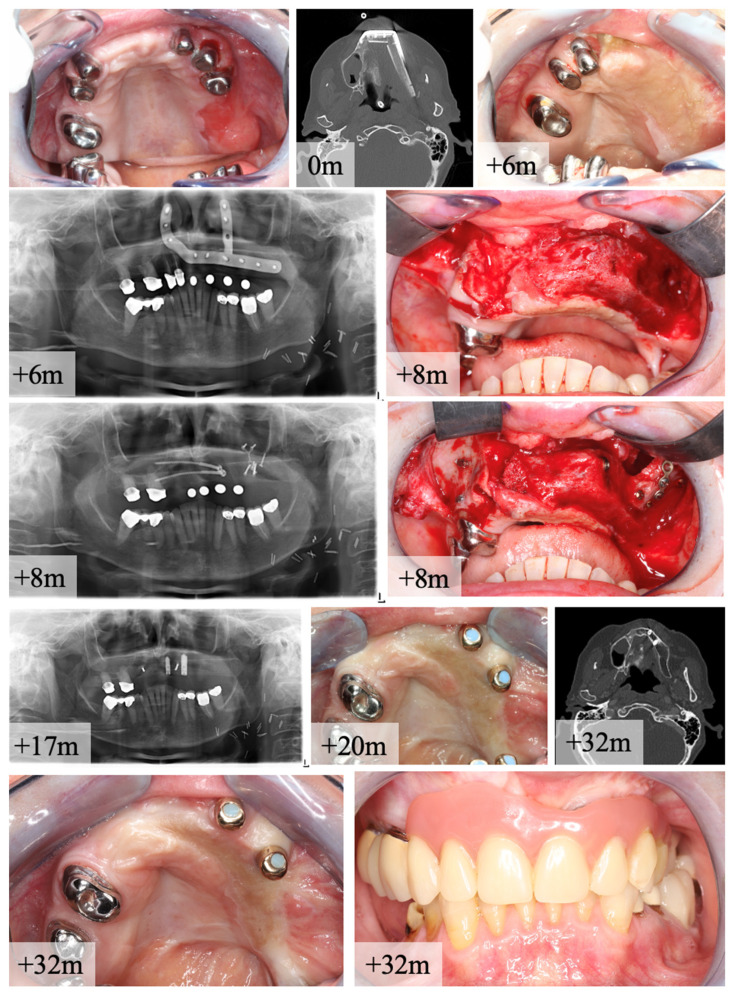
Case of a 65 year-old female patient with oral squamous cell cancer (pT1 pN0 (0/8) L0 V0 cM0) of the alveolar crest in region 13–15. Two-staged reconstruction with bi-segmental composite fibula flap was performed for maxillary bone reconstruction (CT scan shows segments alignment). Re-entry for PSI removal was done after 8 months (m) after reconstruction. OPT shows IOU, but no non-union. Intraoperative finding of a fibrotic joint between maxilla and distal fibula segment. Revision and free bone graft of the iliac crest was used for re-osteosynthesis with two lag-screws and one mini-plate for stabilization. After this procedure, complete bone healing occurred and the insertion of two dental implants was performed. Final CT-scan shows adaption and transformation of the two fibula segments to neo-maxilla. Clinical examination presents stable status of soft tissue and full functional rehabilitation with prosthetic overdenture.

Incomplete osseous union after maxillary reconstruction occurred in 4 patients out of 18 cases after reconstruction with CAD/CAM-PSI. In these cases, bony fusion was found on OPT or CT-scan. After removing the PSI, non-union became clinically visible and revisions with new osteosynthesis and free bone grafts from the iliac crest were necessary. A clinical case is given in Figure 7. The authors assume that the functional stimulus for physiological bone remodeling was not sufficient to achieve primary bone healing when maxillary reconstruction was done with PSI. Gap widths > 1 mm could be excluded in the postoperative CT scans as well as loosening of the osteosynthesis material. Furthermore, during the postoperative consolidation phase, no clinical wound infection was observed at the maxilla-to-fibula junction. In all cases, COU was achieved after revision and repeat osteosynthesis, allowing subsequent implant prosthetic restoration.

Multivariate logistic regression shows that only osteosynthesis type (OR = 3.518 [95%-CI = 1.223–10.124], *p* = 0.02) and adjuvant radiotherapy (OR = 4.804 [95%-CI = 1.602–14.409], *p* = 0.005) are independent risk factors for incomplete osseous union. Adjuvant radiotherapy has been identified as an influencing factor for plate exposure and wound healing disorders [55,56], while other studies do not identify a significant difference [52,57,58].

Limitations

The primary limitations of this study stem from its retrospective approach, specifically the small sample size and different follow-up intervals. Further, we used different types of X-ray examinations, which were not standardized regarding time and type of X-ray examination. Nevertheless, excellent interobserver reliability could be achieved, so assessing the degree of ossification based on these images seems possible. The observed incomplete ossification at the transition zone jaw-to-fibula may be due to adaptation problems during template-guided resection and the PSI’s mechanical (e.g., stiffness) properties. Finally, our study sample includes mono or polysegmental fibula-free flaps for immediate or delayed reconstruction of the maxilla or mandibula of different kinds of primary diseases, which may contribute to a selection bias.

## 5. Conclusions

The study found that complete osseous union was observed slightly earlier in the PSI rather than in the non-PSI group, which is attributed to the small gap width due to the usage of cutting templates. Incomplete bony fusion was significantly more common in the PSI group due to the too-rigid patient-specific osteosynthesis. Jaw-to-fibula apposition zones were significantly more affected than intersegmental zones. Postoperative adjuvant radiotherapy and the usage of PSI-osteosynthesis could be identified as risk-factors for incomplete ossification in multivariate analysis. 

## Figures and Tables

**Table 1:** Study sample characteristics. SD, standard deviation; IQI, interquartile interval; Others: medication-related osteonecrosis of the jaws (*n* = 1), osteoradionecrosis (*n* = 3), osteomyelitis (*n* = 4); * No significant differences were found for maxillary and mandibular reconstruction sites for both osteosynthesis groups.

Parameter	All *n* = 133	Non-PSI *n* = 69	CAD/CAM PSI *n* = 64	*p*-Value
Age (years), mean ± SD	56.7 ± 14.0	56.73 ± 12.11	56.70 ± 15.85	0.990
Follow-up (months), median; IQI (Q1, Q3)	47.0 (21.0, 98.5)	94.0 (63.0, 133.0)	25.5 (16.0, 41.3)	
Gender, *n* (%)				
Male	83 (62.4)	44 (63.8)	39 (60.9)	0.683
Female	50 (37.6)	25 (36.2)	25 (39.1)	
Diagnosis, *n* (%)				
Benign tumor	20 (15.0)	7 (10.1)	13 (20.3)	0.250
Malignant tumor	105 (78.9)	58 (84.1)	47 (73.4)	
Other	8 (6.1)	4 (5.8)	4 (6.3)	
X-ray, *n* (%)				
OPT	347 (60.5)	192 (71.9)	155 (50.5)	0.001
CBCT	41 (7.1)	12 (4.5)	29 (9.4)	
CT	186 (32.4)	63 (23.6)	123 (40.1)	
Reconstruction site: Maxilla, *n* (%)	30 (22.6)	12 (17.4)	18 (28.1)	* 0.139
Uni-segmental	19 (63.3)	8 (66.7)	11 (61.1)	0.757
Bi-segmental	11 (36.7)	4 (33.3)	7 (38.9)	
Reconstruction site: Mandibula, *n* (%)	103 (77.4)	57 (82.6)	46 (71.9)	
Uni-segmental	30 (29.1)	23 (40.4)	7 (15.2)	0.015
Bi-segmental	46 (44.7)	23 (40.4)	23 (50.0)	
Tri-segmental	27 (26.2)	11 (19.3)	16 (34.8)	
Surgery	72 (54.1)	41 (59.4)	31 (48.4)	0.427
Adjuvant radio(chemo)therapy	61 (45.9)	28 (30.6)	33 (51.6)	
Dental status reconstruction site, *n* (%)				
Complete	23 (17.3)	8 (11.6)	15 (23.4)	0.111
Partially edentulous	86 (64.7)	50 (72.5)	36 (56.3)	
Edentulous	24 (18.0)	11 (15.9)	13 (20.3)	
Dental status non-reconstruction site, *n* (%)				
Complete	23 (17.3)	11 (15.9)	12 (18.8)	0.816
Partially edentulous	78 (58.6)	40 (58.0)	38 (59.4)	
Edentulous	32 (24.1)	18 (26.1)	14 (21.9)	

**Table 3:** Multivariate analysis; OR, Odds-Ratio; CI, confidence interval.

Parameter		*p*-Value	OR	95%-CI
Osteosynthesis type	PSI	0.02	3.518	1.223–10.124
Alcohol	Yes	0.08	2.039	0.899–6.486
Adjuvant radiotherapy	Yes	0.005	4.804	1.602–14.409

**Table 4:** Complications are grouped by the used osteosynthesis method and categorized regarding the status of osseous union. Pearson’s χ^2^ or, if n < 5 Fisher exact test was performed. ORN, Osteoradionecrosis.

Complications	Non-PSI *n* = 69		*p*-Value	PSI *n* = 64		*p*-Value
	COU, *n* (%) *n* = 63	IOU, *n* (%) *n* = 6		COU, *n* (%) *n* = 47	IOU, *n* (%) *n* = 17	
Revision and re-Osteosynthesis	4 (6.3)	1 (16.7)	0.375	4 (8.5)	0	0.280
Osteonecrosis	1 (1.6)	0	0.913	0	3 (17.6)	0.016
Screw loosening	9 (14.3)	1 (16.7)	0.624	4 (8.5)	5 (29.4)	0.048
Plate exposure	13 (20.6)	3 (50.0)	0.132	10 (21.3)	5 (29.4)	0.356

## Data Availability

The data presented in this study are available upon request from the corresponding author.
